# P-1751. Discrepancies in Antifungal MIC Values Between VITEK-2 and Etest: Insights from Hospital-Based Fungal Surveillance in Bangladesh

**DOI:** 10.1093/ofid/ofaf695.1922

**Published:** 2026-01-11

**Authors:** Tanzir A Shuvo, Mohammad Abdul Aleem, Nusrat Jahan Shaly, Dilruba Ahmed, Debashis Sen, Asifa Kumkum, Sayeeda Huq, Fahmida Chowdhury

**Affiliations:** icddr,b, Dhaka, Dhaka, Bangladesh; icddr,b, Dhaka, Dhaka, Bangladesh; icddr,b, Dhaka, Dhaka, Bangladesh; icddr,b, Dhaka, Dhaka, Bangladesh; icddr,b, Dhaka, Dhaka, Bangladesh; icddr,b, Dhaka, Dhaka, Bangladesh; icddr,b, Dhaka, Dhaka, Bangladesh; icddr,b, Dhaka, Dhaka, Bangladesh

## Abstract

**Background:**

Invasive fungal infections (IFIs) are associated with severe complications and high mortality, particularly among critically ill patients. Despite their clinical importance, the true burden of IFIs remains poorly understood in low- and middle-income countries (LMICs) such as Bangladesh. While species identification is essential, antifungal susceptibility testing (AFST) plays a more critical role in informing appropriate clinical management. This study aimed to compare the minimum inhibitory concentration (MIC) values generated by the VITEK 2 automated system with those obtained using the Etest method.

**Figure:**
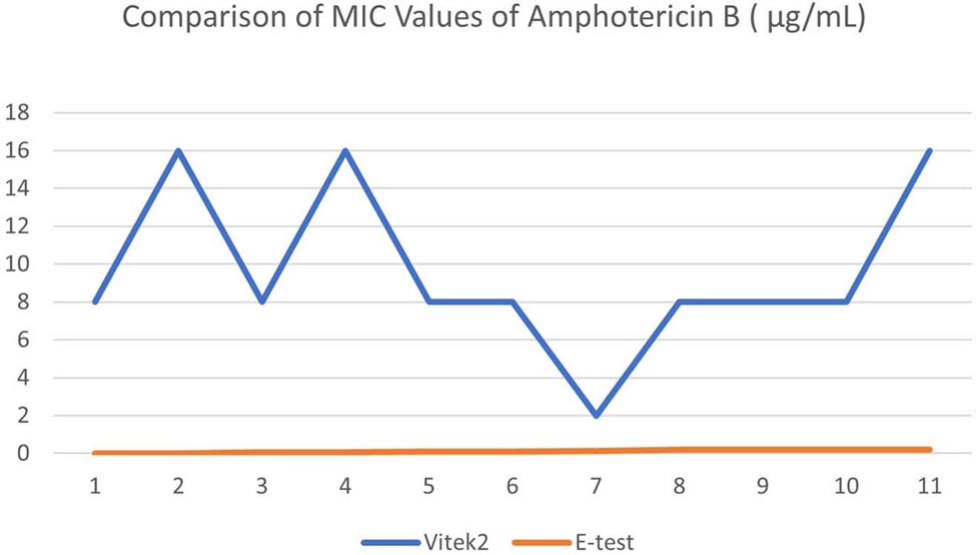
Comparison of MIC Values of Amphotericin B ( µg/mL) Comparison of MIC Values of Voriconazole ( µg/mL)

**Figure:**
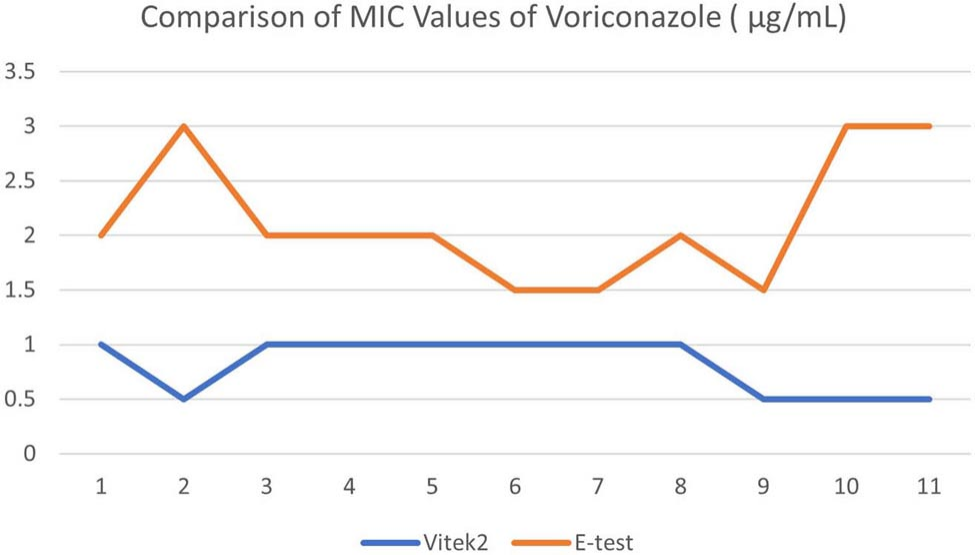

**Methods:**

Eleven blood isolates of Candida ciferrii were identified, and MIC values for amphotericin B and voriconazole were initially obtained using the VITEK 2 automated system. The isolates were preserved at –80°C and later revived at SDA (sabouraud dextrose agar) for Etest-based susceptibility testing. Etest strips (AMPHOTERICIN B AP M100 WW and VORICONAZOLE VO M100 WW) were applied to dry RPMI agar plates. The inoculum was standardized to a McFarland range of 0.50–0.63 using a DensiChek device. Plates were incubated aerobically at 37°C for 18–24 hours before MIC values were recorded.

**Results:**

Significant discrepancies were observed between the minimum inhibitory concentration (MIC) values obtained using the VITEK-2 automated system and the Etest method. For *Amphotericin B*, the mean MIC generated by the VITEK-2 system was 9.64 µg/mL (median: 8), which was substantially higher than the mean MIC of 0.11 µg/mL (median: 0.09) obtained through the Etest method. In contrast, for *Voriconazole*, the mean MICs were 0.82 µg/mL (median: 1) via VITEK-2 and 2.14 µg/mL (median value not reported) via Etest.

**Conclusion:**

These findings highlight considerable variation between the two methods, raising concerns regarding the reliability and clinical interpretability of automated susceptibility results. Such inconsistencies can hinder evidence-based antifungal therapy. The surveillance team is currently conducting additional tests to validate these observations and establish a clearer understanding. Further research is essential to address these methodological differences and ensure consistent and accurate antifungal susceptibility testing in clinical settings.

**Disclosures:**

All Authors: No reported disclosures

